# 

**Published:** 1886-04

**Authors:** 


*Entered at the Post Office, at Austin, Texas, as Second-class Matter.*

VOL. 1.] **APRIL, 1886.** [No. 10.

LIBRARY
INDEXED
S.G.O.

### DANIEL'S MEDICAL JOURNAL

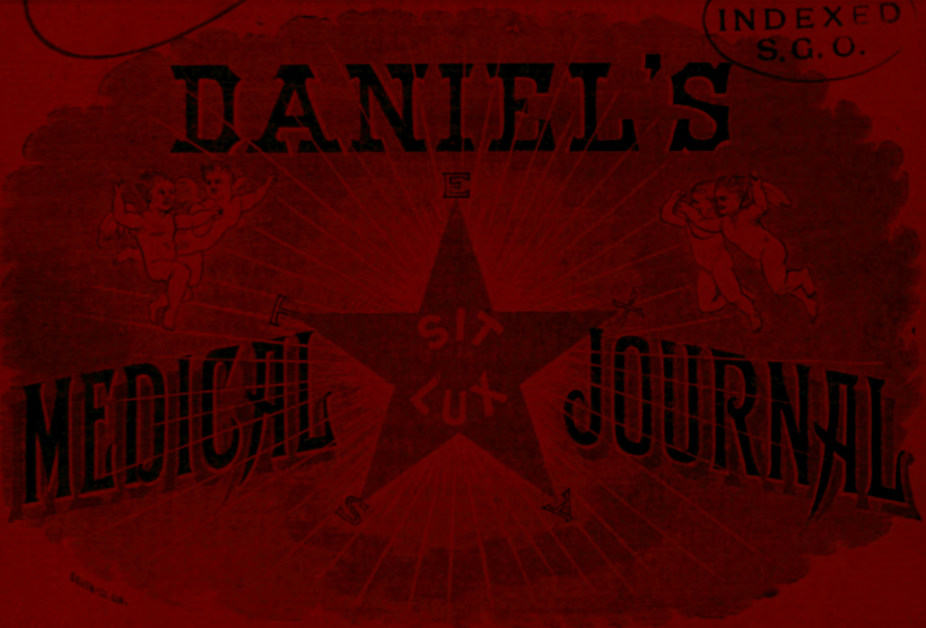

*Subscription: $2.00 a Year in Advance; - - - Single Copies, 25 Cents*

F. E. DANIEL, M. D., Editor and Publisher, AUSTIN, TEXAS.

NORTHERN OFFICE, 1217 FILBERT STREET, PHILADELPHIA.

*EUGENE VON BOECKMANN, PRINTER, AUSTIN, TEXAS.*

